# Targeting the Retinoid X Receptor Pathway Prevents and Ameliorates Murine Chronic Graft-Versus-Host Disease

**DOI:** 10.3389/fimmu.2022.765319

**Published:** 2022-03-11

**Authors:** Govindarajan Thangavelu, Michael C. Zaiken, Fathima A. Mohamed, Ryan Flynn, Jing Du, Stephanie Y. Rhee, Megan J. Riddle, Ethan G. Aguilar, Angela Panoskaltsis-Mortari, Martin E. Sanders, Bruce R. Blazar

**Affiliations:** ^1^ Department of Pediatrics, Division of Blood & Marrow Transplantation & Cellular Therapy, University of Minnesota, Minneapolis, MN, United States; ^2^ Io Therapeutics Inc., Santa Ana, CA, United States

**Keywords:** RXR, TFH, TfR, IL-17, germinal center B cells

## Abstract

Most allogeneic hematopoietic stem cell transplant (allo-HSCT) recipients receive peripheral blood stem cell grafts resulting in a 30%–70% incidence of chronic graft-versus-host disease (cGVHD), a major cause of mortality and morbidity in long-term survivors. While systemic steroids remain the standard of care for first-line therapy, patients may require long-term administration, and those with steroid-resistant or refractory cGVHD have a worse prognosis. Although durable and deep responses with second-line therapies can be achieved in some patients, there remains an urgent need for new therapies. In this study, we evaluated the efficacy of IRX4204, a novel agonist that activates RXRs and is in clinical trials for cancer treatment to prevent and treat cGVHD in two complementary murine models. In a major histocompatibility complex mismatched, non-sclerodermatous multiorgan system model with bronchiolitis obliterans, IRX4204 prevented and reversed cGVHD including associated pulmonary dysfunction with restoration of germinal center T-follicular helper: T-follicular regulatory cell balance. In a minor histocompatibility antigen disparate sclerodermatous model, IRX4204 treatment significantly prevented and ameliorated skin cGVHD by reducing Th1 and Th17 differentiation due to anti-inflammatory properties. Together, these results indicate that IRX4204 is a promising therapeutic option to treat cGVHD with bronchiolitis obliterans or sclerodermatous manifestations.

## Introduction

Chronic graft-versus-host disease (cGVHD) is a life-threatening complication following allogeneic hematopoietic stem cell transplantation (allo-HSCT), which causes late non-relapse morbidity and mortality (1, 2). Although recent studies have advanced the understanding of GVHD pathophysiology, the first-line therapy remains corticosteroids, which can achieve a complete response in only 20%–50% of patients (3, 4). For patients that do not respond to steroid therapy, the mortality rate is high (5). Furthermore, allo-HSCT recipients undergoing broad immunosuppressant therapy are more prone to tumor relapse, infections, and drug-related toxicities. Therefore, novel targeted immunomodulatory strategies are highly warranted to improve clinical outcomes in allo-HSCT.

Chronic GVHD patients with either bronchiolitis obliterans (BO), an obstructive pulmonary disease, or scleroderma complications are poor responders to standard available therapies (6–9). Moreover, complete and durable responses in steroid refractory or dependent cGVHD are infrequent. Hence, multiple preclinical cGVHD models have been developed to represent aspects of the spectrum of cGVHD manifestations and further elucidate cGVHD pathogenesis in an attempt to develop therapeutic strategies (10–15). Since cGVHD with BO has a poor prognosis, a cGVHD BO model was developed as a platform to evaluate new therapies. Toward that end, B10.BR (H2^k^) recipients were conditioned with high-dose cyclophosphamide and total body irradiation prior to reconstitution with major histocompatibility complex (MHC)-disparate C57BL/6 (H2^b^) bone marrow and a low dose of T cells, recapitulating many of the clinical, functional, and pathological manifestations of cGVHD with BO (13, 16). Chronic GVHD pathogenesis with BO was dependent upon alloreactive donor CD4+ T cells that differentiated into T-follicular helper (Tfh) cells to activate germinal center (GC) B cells, resulting in pathogenic antibody production and disposition onto cGVHD target organs. Monocyte and macrophage recruitment into areas of lung injury results in stimulation of fibroblast release of profibrogenic molecules and fibrosis in target organs except the skin (17). In a minor histocompatibility antigen disparate scleroderma model, BALB/c (H2^d^) recipients underwent total body irradiation prior to reconstitution with B10.D2 (H2^d^) bone marrow and T cells resulting in fibrotic skin disease mediated by donor T helper 1 (Th1) and Th17 cells (18).

Retinoid X receptors (RXRs) are key members of the nuclear receptor (NR) superfamily due to their diverse roles in modulating various physiological processes (19). RXRs form homodimers (RXR-RXR) and heterodimers with other NRs, namely, retinoic acid receptors (RARs), thyroid hormone receptor, liver X receptors (LXRs), vitamin D receptor, farnesoid x receptor, nuclear receptor related 1 protein (Nurr1, Nr4a2), nerve growth factor IB (Nur77, Nr4a1), and peroxisome proliferator-associated receptors (PPARs) (20). Studies in mice have reported the direct role of RXR in controlling Th1 differentiation with loss of RXRα in CD4+ T cells leading to increased Th1 polarization and interferon gamma (IFN-γ) production (21). Since RXRα is also required to maintain the suppressive function of T regulatory cells (Tregs), RXR agonists could have dual benefits as a therapeutic strategy for controlling inflammatory disorders.

Rexinoids are RXR ligands that selectively bind and activate RXRs. Although rexinoids can have high therapeutic value in treating various metabolic disorders and cancers (22, 23), off-target responses due to RXR promiscuity with NRs other than RARα limited their clinical applications (24–26). For instance, in addition to RARα, Food and Drug Administration (FDA)-approved synthetic rexinoid bexarotene binds with liver-X-receptor (LXR) and PPAR (27–29). IRX4204 is a highly selective rexinoid with a potency of 100-fold more than bexarotene in activating RXRs. Specifically, IRX4204 does not transactivate RXR heterodimers of RAR, LXR, or PPAR (30), consistent with the selective biological function of IRX4204 and pointing to its use as a therapeutic agent. Recent studies have demonstrated the therapeutic efficacy of IRX4204 in controlling murine autoimmune disease and acute GVHD (aGVHD) (20, 31). Administration of IRX4204 in a murine model of multiple sclerosis attenuated the severity of the disease (20). We previously showed that IRX4204 treatment ameliorated aGVHD while retaining graft-versus-tumor (GVT) responses against leukemia and lymphoma cells (31). Since the pathophysiology of cGVHD is distinct from aGVHD, we sought to test the prophylactic and therapeutic efficacy of IRX4204 in two pathologically distinct cGVHD models. Herein, we demonstrate that IRX4204 administration prevents and treats both BO and sclerodermatous manifestations of cGVHD. Mechanistically, IRX4204 impairs pathogenic donor Tfh, Th1, and Th17 differentiation leading to protection from cGVHD.

## Materials and Methods

### Mice

Female C57BL/6 (B6;H2^b^) and BALB/c (H2^d^) mice were purchased from the National Cancer Institute. Female B10.BR (H2^k^) and B10.D2 (H2^d^) mice were purchased from The Jackson Laboratory. All mice ranged in age from 10 to 18 weeks. Mice were housed in a specific pathogen-free facility, and all studies were approved by the University of Minnesota’s Institutional Animal Care Committee.

### BM Transplantation

For the BO model, B10.BR recipients were conditioned with cyclophosphamide on days −3 and −2 (120 mg/kg/day intraperitoneally). On day −1, recipients received total body irradiation (TBI) by X-ray [8.3 Gray (Gy) by X-ray)]. Recipients then received 10 × 10^6^ B6 T-cell-depleted (TCD) bone marrow (BM) only or with 7–7.5 × 10^4^ purified splenic T cells on day 0 (17, 32). In the scleroderma model, BALB/c mice were given lethal TBI (7 Gy by X-ray, day −1) and 10^7^ B10.D2 TCD-BM only or with 1.8 × 10^6^ CD4^+^ and 0.9 × 10^6^ CD8 T cells (day 0) (32, 33). Mice were monitored daily for survival. Skin scores were assessed twice weekly (32).

### Pulmonary Function Tests

Pulmonary function tests (PFTs) were performed as previously described (16). Briefly, mice were anesthetized with Nembutal, intubated and ventilated using the Flexivent system (Scireq Montreal, QC). Pulmonary resistance, elastance, and compliance were reported using Flexivent software version 7. Chronic GVHD controls have increased pulmonary resistance and elastance along with decreased compliance as compared to BM-only controls in our BO cGVHD model (13, 16).

### Flow Cytometry

For Tfh and GC B cells, single-cell suspensions of spleens were obtained and stained with fluorochrome-labeled anti-CD4 (RM4-5, BD), anti-CXCR5 (SPRCL5, Thermo Fisher Scientific, MA, USA), anti-PD-1 (J43, Thermo Fisher Scientific), anti-CD19 (eBio1D3, Thermo Fisher Scientific), anti-GL7 (GL-7, Thermo Fisher Scientific), and anti-Fas (J02, BD). Live/dead fixable viability dye (Thermo Fisher Scientific) was used for live/dead discrimination. GC B cells were defined as Fas and GL7 double-positive CD19 B cells. Tfh cells were defined as PD1 and CXCR5 high CD4+ Foxp3− T cells. Tfr cells were defined as PD1, and CXCR5 high CD4+ Foxp3+ T cells. Tregs were detected by staining cells for surface antigens, followed by fixation, permeabilization using a Foxp3/transcription factor staining buffer set (Thermo Fischer Scientific), and labeling with anti-Foxp3 (FJK-16s, Thermo Fisher Scientific). For intracellular cytokine staining experiment, lymph node and spleen cells were stimulated with cell stimulation cocktail (plus protein transport inhibitors) (Thermo Fisher Scientific) for 5 h at 37°C. After surface staining, cells were fixed, permeabilized, and stained with anti-interleukin-17 (anti-IL-17) (eBio17B7, Thermo Fisher Scientific) and anti-IFN-γ (XMG1.2, Thermo Fisher Scientific). BD LSRFortessa (BD Biosciences, CA, USA) was used to acquire cells, and analyses were performed using FlowJo software.

### Immunofluorescence

For the GC detection, acetone-fixed 6-µm cryosections of spleens were stained with rhodamine-peanut agglutinin (Vector Laboratories, CA, USA). For CD4 and B-cell staining, sections were stained with CD4 FITC (RM4-5, Thermo Fisher Scientific) and B220 BV421 (RA3-6B2, BD). GCs are identified as PNA+ regions with B220+ and/or CD4+ areas surrounding them. Confocal images were acquired on an Olympus FluoView500 Confocal Laser Scanning Microscope at 200×, analyzed using FluoView3.2 software (Olympus), and quantified using a Voronoi tessellation methodology making use of EBImage (34).

### NP-OVA Immunizations

B6 mice were immunized with 4-hydroxy-3-nitrophenylacetyl hapten (NP)-OVA (100 μg, Bioresearch Technologies, Novato, CA, USA), diluted in complete H37 Ra (Becton, Dickinson and Company, Franklin Lakes, NJ, USA) in each flank (35). For IRX4204 prophylaxis, mice were given IRX4204 on days 0–7 daily (i.p.), then sacrificed 7 days post-immunization, and the inguinal lymph nodes were harvested and analyzed by flow cytometry. For therapeutic purposes, mice were treated with IRX4204 on days 8–14 daily (i.p.) 7 days post-immunization. On day 14, inguinal lymph nodes were harvested and analyzed by flow cytometry.

### IRX4204

IRX4204 (Io Therapeutics, USA) was prepared in phosphate-buffered saline (PBS) [containing ~4% dimethyl sulfoxide (DMSO) and 1% Tween 80], once in a week and stored at 4°C (36). Chronic GVHD recipients were given vehicle or IRX4204 daily at a dose rate of 200 μg/mouse i.p as indicated.

### Histopathology and Trichrome Staining

Tissue sections were embedded in optimal cutting temperature (OCT) compound, snap-frozen in liquid nitrogen, and stored at −80°C. Lungs were inflated by 75% OCT before harvest and freezing. Acetone-fixed 6-μm cryosections were hematoxylin and eosin stained and evaluated as described (37). For trichrome staining, 6-μm cryosections were fixed for overnight in Bouin’s solution and stained with the Masson’s trichrome staining kit (Sigma HT15) for detection of collagen deposition.

### Statistical Analysis

GraphPad Prism 7 was used to conduct statistical analysis. One-way ANOVA with Bonferroni correction and Student’s t-test were used for statistical analysis as indicated. Error bars indicate mean ± standard error mean (significance: *p <.05; **p <.01; ***p <.001; ****p <.0001).

## Results

### IRX4204 Prevents and Reverses BO cGVHD

**Figure 1:**
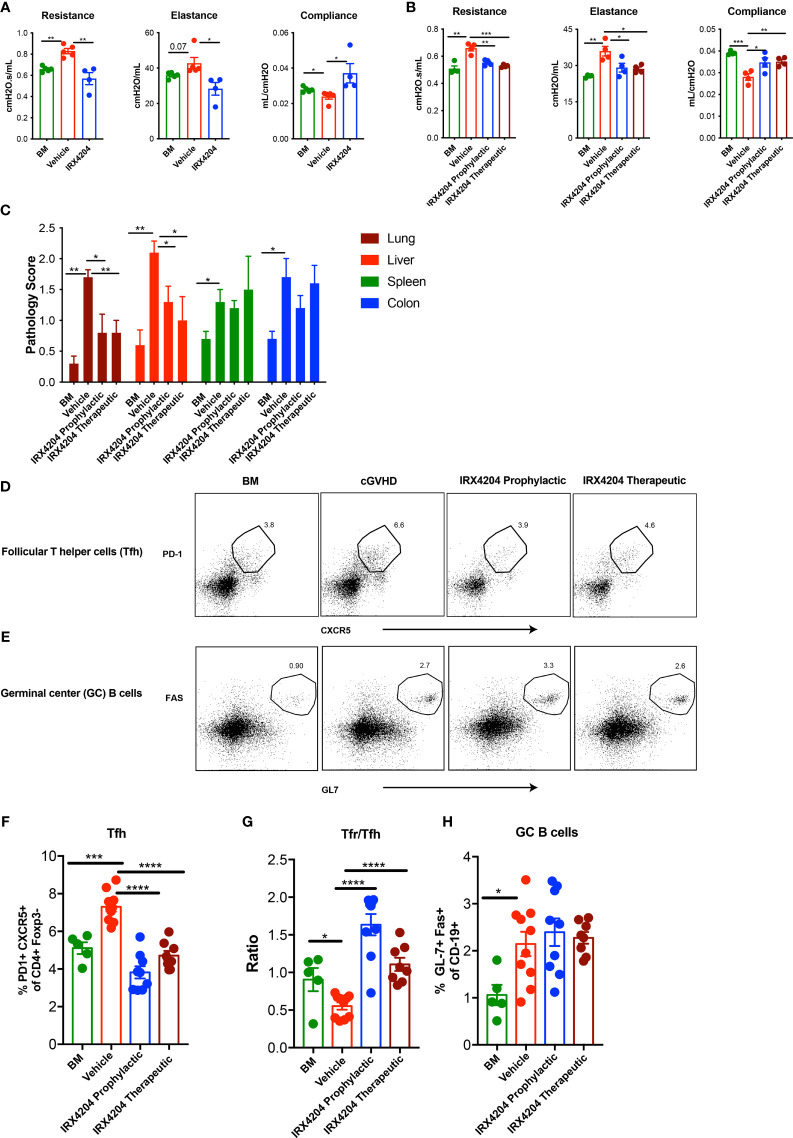
IRX4204 prevents and reverses established cGVHD-mediated BO. Conditioned B10.BR mice were transplanted with B6 donor BM ± T cells. A cohort of BM + T recipients were treated with IRX4204 either from days 0 to 28, or 28 to 56. Pulmonary tests including lung resistance, elastance, and compliance were performed on **(A)** day 28 and **(B)** day 56 post-transplantation n = 4 – 5/group. **(C)** Histopathology scores of hematoxylin and eosin–stained tissue sections from lung, liver, spleen, and colon on recipient on day 58. n = 5/group. Representative flow plots of **(D)** Tfh and **(E)** GC B cells. Frequency of Tfh **(F)**, Ratio of Tfr/Tfh **(G)** and frequency of GC B cells **(H)** in recipient spleen on day 58. *p <.05; **p <.01; ***p <.001; ****p <.0001. Error bars represent standard error of the mean (SEM); n = 5 - 10 per group.

To evaluate the prophylactic efficacy of IRX4204 in cGVHD, we utilized a major MHC mismatched, murine multiorgan system model of cGVHD with BO. In this model, B10.BR recipients were preconditioned with cyclophosphamide and TBI followed by transplantation with donor B6 TCD BM alone or TCD-BM plus a low dose of T cells (13, 16). We previously reported that the loss of pulmonary function due to fibrotic change in the lung was detected as early as day 28 (13). Using a FlexiVent (SCIREQ) system, we performed PFTs on day 28 in cGVHD recipients treated with either vehicle or IRX4204. Recipients receiving IRX4204 daily from day 0 to 28 had significantly improved pulmonary function compared to those given vehicle (**Figure 1A**). These studies showed that cGVHD with BO was established by day 28 as demonstrated by PFTs and that IRX4204 given days 0–28 was sufficient to prevent cGVHD onset. Furthermore, mice given prophylactic IRX4204 showed improved PFTs by day 56 as compared to control recipients (**Figure 1B**).

We next sought to determine whether IRX4204 could reverse the established cGVHD by treating cGVHD mice beginning on day 28. On day 56, cGVHD mice that were continuously treated from day 28 showed improved PFTs as compared to vehicle-treated cGVHD recipients and comparable to BM-only recipients (**Figure 1B**) Consistent with the reduced cGVHD clinical signs, pathology scores of lungs and liver were significantly lower in recipients of either prophylactic or therapeutic IRX4204 as compared to vehicle controls (**Figure 1C**). No significant changes were observed in the spleen and colon of mice that received IRX4204.

Increased Tfh and GC B cells can promote while Tfrs can inhibit BO cGVHD pathogenesis by controlling GC formation and allo- or auto-antibody production and deposition in cGVHD target tissues (17, 38). To determine whether IRX4204 treatment induces immune cell changes that could preclude or disrupt GC formation in cGVHD mice, we analyzed the frequency of Tfh, Tfrs, and GC B cells in day 58 post-BMT spleens of recipients treated daily with IRX4204 from day 0 to 28 or day 28 to 56. IRX4204-treated mice had a reduced frequency of pathogenic Tfh with an increased frequency of immunoregulatory Tfrs, resulting in elevated Tfr/Tfh ratios (**Figures 1D, F, G**). Cumulatively, these data demonstrate that IRX4204 treatment is effective at preventing and reversing cGVHD with BO as measured by PFTs, lung and liver pathology scores, and Tfr/Tfh imbalance associated with diminution of the GC immune responses.

### IRX4204 Impairs GC Reactions

**Figure 2:**
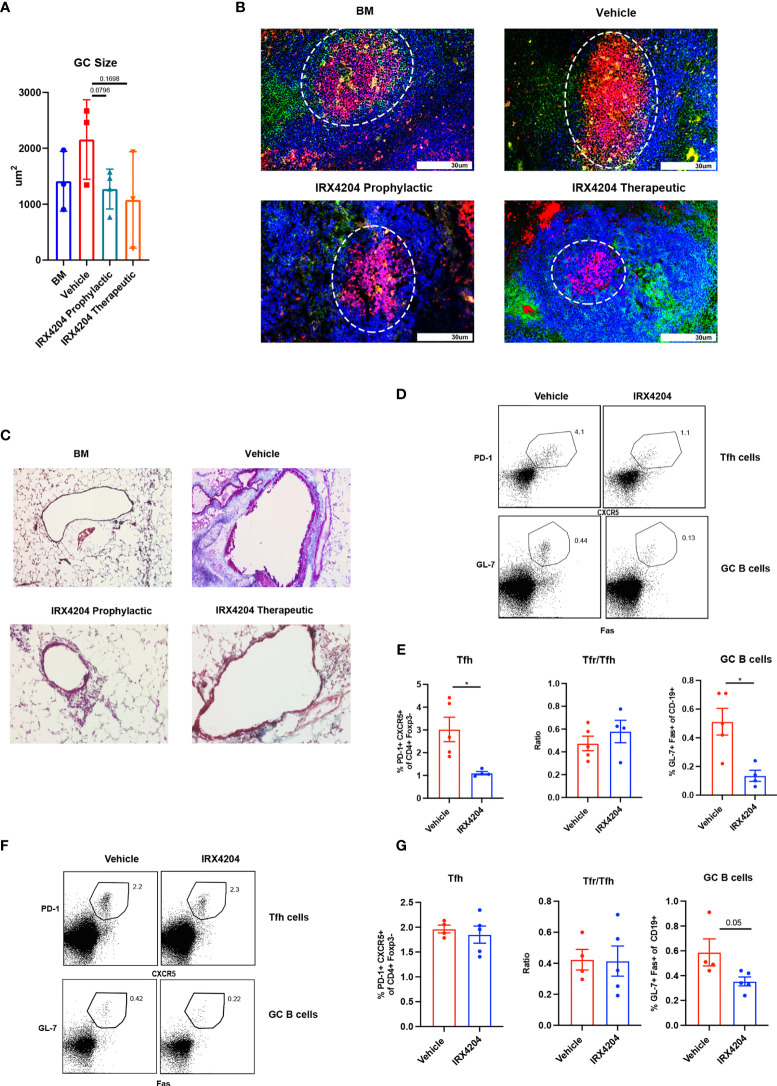
IRX4204 impairs GC B cell response in an immunization model but not in cGVHD. **(A, B)** Conditioned B10.BR mice were transplanted with B6 donor BM ± T cells. A cohort of BM + T recipients were treated with IRX4204 either from days 0 to 28, or days 28 to 56. n = 3 mice/group. **(A)** GC size and **(B)** representative splenic GC immunofluorescence images from BM only and BM plus T-cell mice on day 58 showing peanut agglutinin (PNA; Red). CD4 FITC (green) and B220 BV421 (blue). Germinal centers are highlighted in white circle. An Olympus FluoView500 confocal laser scanning microscope was used to acquire images at magnification 200×. **(C)** Representative images of Masson’s trichrome staining. Collagen was identified as the area stained in blue. EVOS XL Imaging system was used to acquire images at magnification 200×. **(D–G)** WT mice were immunized with NP-OVA and treated with either vehicle or IRX4204 daily. **(D, E)** Flow plots of Tfh, GC B cells, and quantification of GC B cells, Tfh, and Tfr/Tfh ratio from inguinal draining lymph nodes 7 days post-immunization. IRX4204 or vehicle was given from day 0 to 7 (prophylactic). **(F, G)** Flow plots of Tfh, GC B cells, and quantification of GC B cells, Tfh, and Tfr/Tfh ratio from inguinal draining lymph nodes 14 days post-immunization. IRX4204 or vehicle was given from day 7 to 14 (therapeutic). *p < .05. Error bars represent standard error of the mean (SEM); n = 4/group.

Although the GC B-cell frequency was not significantly different between the groups (**Figures 1E, H**) despite reduced Tfh and increased Tfr and Tfr/Tfh ratio, there was a statistical trend towards smaller GC size in recipients of IRX4204 prophylaxis (**Figures 2A, B**). Diminished GC size is consistent with the known cause–effect relationship between high GC numbers and size and cGVHD with BO (13, 17). Peri-bronchiolar collagen deposition is a characteristic feature of BO-cGVHD (13). We performed Masson’s trichrome staining to identify collagen in tissue section. Compared to vehicle controls, mice treated with IRX4204 either as prophylaxis or therapy had a decrease in the accumulation of collagen in the lungs around the bronchioles (**Figure 2C**).

To determine IRX4204 effects on GC reactions due to nominal antigen delivered in adjuvant in a non-cGVHD setting, wild-type mice were immunized with NP-OVA, emulsified in complete H37 Ra. One cohort of mice was treated daily with IRX4204 as prophylaxis for GC formation beginning on the day of immunization. A second cohort was treated with IRX4204 beginning after GC formation beginning on day 7 after immunization. After 7 days of IRX4204 administration, draining lymph nodes were harvested and the frequency of Tfh and GC B cells assayed. IRX4204 treatment as either prophylaxis (**Figures 2D, E**) or therapy (**Figures 2F, G**) significantly inhibited the induction of GC B cells compared to the vehicle treatment. However, we found that IRX4204 had the ability to reduce Tfh induction only when administered prophylactically (**Figures 2D, E**). Despite reduced Tfh in mice given IRX4204 prophylaxis, Tfr/Tfh ratios were unchanged. Not surprisingly, as Tfh were not reduced by IRX4204 given as a therapeutic agent, Tfr/Tfh ratio did differ between IRX4204 and vehicle (**Figures 2F, G**).

### IRX4204 Is Effective as Prophylaxis and Therapy for Sclerodermatous cGVHD

**Figure 3:**
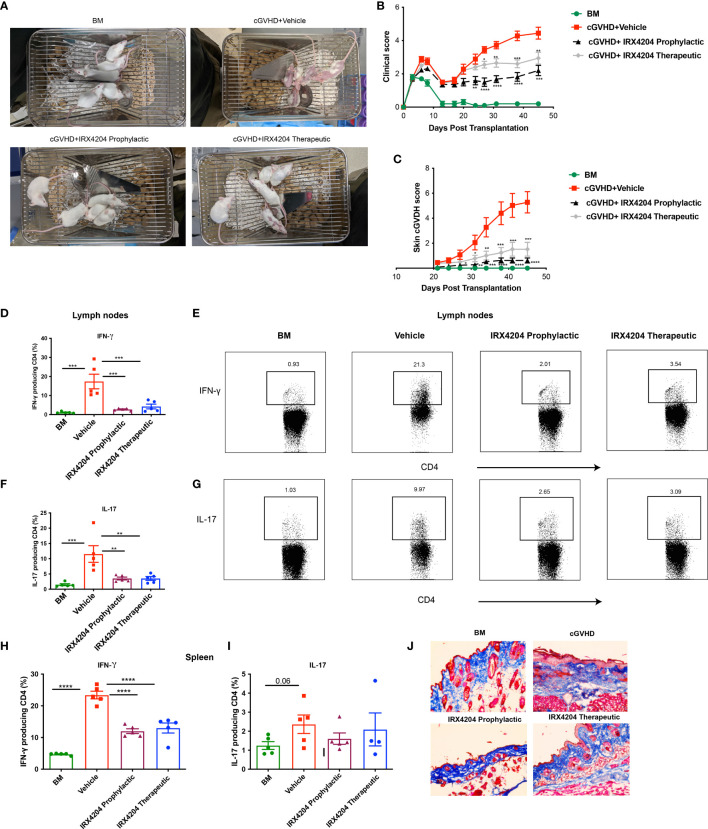
IRX4204 inhibits and treats sclerodermatous cGVHD. Lethally irradiated BALB/c mice were transplanted with B10.D2 BM only or with purified T cells (CD4, CD8: 1.8 x 10^6^ and 0.9 x 10^6^, respectively). Recipients were treated with IRX4204 either from day 0-21 (IRX4204 prophylactic) or day 21 (IRX4204 therapeutic). **(A)** Photographs of mice in BM, cGVHD + vehicle or IRX4204 treated cGVHD groups. **(B)** Clinical manifestations of cGVHD were assessed by giving scores to weight loss, activity, posture and fur condition. Healthy mice receive score 0. **(C)** Skin scores were assessed by measuring the area of skin with fur loss or sclerodermatous lesion. Intact skin was given a score of 0. **(D–I)** Peripheral lymph nodes (LNs) and spleens (SPL) were harvested on day 50 post-transplantation and stimulated with PMA and ionomycin *in vitro*. **(D–G)** The percentages and representative flow plots of **(D–E)** IFN-γ+ and **(F, G)** IL-17+ producing CD4+ T cells in LNs are shown. **(H, I)** The percentages of **(H)** IFN-γ+ and **(I)** IL-17+ producing CD4+ T cells in SPL are shown. **(J)** Trichrome staining of skin. Collagen was stained in blue. *p < .05; **p < .01; ***p < .001; ****p < .0001. Error bars represent standard error of the mean (SEM); n = 8 – 10 per group.

Having demonstrated the potency of IRX4204 in attenuating cGVHD in the BO model that lacks skin manifestations, we next evaluated its efficacy in a classical model of sclerodermatous cGVHD. BALB/c recipients were lethally irradiated and transplanted with multiple minor mismatched B10.D2 donor BM ± T cells. In this model, cutaneous manifestations become apparent at approximately day 21 (39, 40). We tested the prophylactic and therapeutic efficacy of IRX4204 starting treatment from either day 0 or day 21 for a period of 50 days. We found that the administration of IRX4204 either as prophylaxis or therapy significantly improved cGVHD clinical and skin scores compared to vehicle controls (**Figures 3A–C**).

Sclerodermatous cGVHD is mediated by donor CD4+ T cells producing IFN-γ and IL-17 (18). To explore the cellular mechanisms associated with cGVHD protection in IRX4204-treated recipients, we examined the frequency of IFN-γ+ and IL-17+ producing CD4+ T cells in recipient peripheral lymph nodes and spleens. On day 50, compared to control, we found that IRX4204 treatment, both prophylactic and therapeutic, significantly reduced the frequency of CD4+ T cells producing IFN-γ and IL-17 in peripheral lymph nodes (**Figures 3D–G**). In addition, the frequency of splenic CD4+ IFN-γ+ but not IL-17 producing T cells was significantly reduced in the spleens of mice given IRX4204 as prophylaxis or therapy (**Figures 3H, I**). To evaluate whether IRX4204 reduces collagen deposition on skin, serially sectioned skin tissues were stained with Masson’s trichrome stain. Compared to vehicle controls, mice treated with IRX4204 either as prophylaxis or therapy had a decrease in the accumulation of collagen in the skin (**Figure 3J**). Taken together, IRX4204 alleviated sclerodermatous cGVHD associated with reduced Th1 and Th17 differentiation in secondary lymphoid organs.

## Discussion

Identification of novel immunomodulatory therapies to prevent and treat cGVHD remains the unmet clinical need in allo-HSCT. In the current study, we demonstrated that a highly potent rexinoid, IRX4204, prevented and treated cGVHD by impairing pathogenic donor T-cell responses in two preclinical models with distinct pathophysiology. Therefore, targeting the RXR pathway with IRX4204 is a promising therapeutic strategy to reduce cGVHD. Furthermore, IRX4204 impairs GC B-cell generation in mice immunized with NP-OVA.

In the MHC mismatched BO model, IRX4204 treatment prevented and reversed established cGVHD as reflected by improved PFTs. Although we found that IRX4204 was effective in reducing the cGVHD-mediated lung and liver pathology, there was no effect on spleen and colon pathology. The lack of effect may be due to contrasting mechanisms of cGVHD in different organs. We previously demonstrated that Tfh, a subset of CD4+ T cells, plays a key role in promoting BO cGVHD pathogenesis by supporting GC B-cell affinity maturation and differentiation to produce pathogenic antibodies (17). An earlier study reported that RXR activation has been shown to negatively affect Tfh differentiation in a chronic model of inflammation (41). A more recent study reported that RARα signaling enhances Tfh differentiation in an airway inflammation mouse model (42), perhaps through inhibiting IL2Rα upregulation on T cells, as IL2R signaling inhibits Tfh differentiation by repressing BCL6. In the current study, we reasoned that IRX4204 would modulate Tfh differentiation in cGVHD mice by directly impairing donor T-cell responses. Indeed, we found that IRX4204 treatment reduced the frequency of pathogenic Tfh cells, consistent with the known direct suppressive effect on the proliferation of murine and human T cells *in vitro* (31) and *in vivo* in IRX4204-treated aGVHD mice (31).

Chronic GVHD patients have a reduced frequency of Tregs (43), which leads to the lack of functional tolerance and subsequent immune dysregulation. Restoring immune tolerance through either expansion of Tregs *in vivo* or adoptive Treg transfer has been shown to be an effective strategy to reduce cGVHD (44–46). Here, we show the increased frequency of Tfrs when recipient mice were treated with IRX4204 compared with vehicle-treated recipients. IRX4204 is a potent activator of Nr4a2, which has been shown to promote Treg generation and stability (47). Consistent with the Nr4a2 activity, IRX4204 fostered *in vitro* induced Treg (20, 31) and pTreg generation in aGVHD mice (31).

Studies have shown that Tfrs impair the production of pathogenic antibodies by negatively regulating Tfh and GC B-cells responses (38, 48). Therefore, it is likely that both increased Tfr frequency and Tfr/Tfh ratios in IRX4204-treated mice contribute to the alleviated cGVHD. Furthermore, this cGVHD/BO model depends upon Tfh production of IL-21. IL-21 is counterregulatory to pTreg production, as we reported in an aGVHD model (49), and Treg function (50, 51). Taken together, increased Tfr in IRX4204-treated mice may be due to Nr4a2 pathway activation in donor T cells resulting in suppression of Tfh IL-21 production, fostering Treg suppressor function and the generation of Tregs and Tfrs over Tfhs. By employing the NP-OVA immunization model, we observed that prophylactic IRX4204 reduced the frequency of Tfh and GC B cells, although unexpectedly, we did not observe any significant differences in GC B cells between IRX4204 and vehicle-treated mice in cGVHD/BO mice. However, compared to vehicle-treated mice, IRX4204-prophylactically treated mice had a statistical trend toward smaller GC size.

Scleroderma is a serious and severe fibrosing disorder that occurs in the majority of cGVHD patients affecting the skin, subcutaneous tissue, and fascia (52). In an MHC-matched, multiple minor antigen mismatched scleroderma mouse model (32, 53), IRX4204 treatment as either a prophylactic or therapeutic significantly reduced sclerodermatous cGVHD with improved clinical outcomes. A previous study showed that in a scleroderma cGVHD model, transplantation of either donor IL-17^−/−^ or IFN-γ^−/−^ T cells significantly ameliorated the disease (18). In the current study, we observed a reduced frequency of Th1 and Th17 cells in IRX4204-treated recipients, consistent with previous studies showing that IRX4204 impaired Th1 and Th17 differentiation in aGVHD and experimental autoimmune encephalomyelitis, respectively (20, 31). RA signaling is required to maintain the stability of Th1 cells (54). We and others demonstrated that heightened RA synthesis during allo-HSCT exacerbated aGVHD lethality by enhancing Th1 differentiation of donor T cells (55–57). IRX4204 may directly suppress Th1 differentiation *via* Nr4a2 activation, as Nr4a2 has been shown to repress Th1 lineage commitment (47) or as a result of inadequate RA signaling in donor T cells.

Although RA signaling has been shown to exacerbate aGVHD (55–57), Nishimura and colleagues (18) demonstrated that the *in vivo* administration of synthetic retinoid attenuated scleroderma cGVHD by reducing the differentiation of Th1 and Th17. Since IRX4204 preferentially activates RXR homodimers, the endogenous RA signaling pathway that is heightened in GVHD may be impaired in cGVHD mice due to the competitive binding of RXRs to the agonist or other receptors, reducing binding with RARs. Whereas the RXR agonist tributyltin inhibited Th17 differentiation that mechanistically may be the consequence of LXR-RXR pathway activation (58), IRX4204 inhibition of Th17 differentiation *in vitro* and *in vivo* (20) does not require LXR activation, suggesting that RXR homodimers or other RXR binding partners are involved in controlling Th17 differentiation. Notably, IL-17 is known to support GC formation, ectopic lymphoid follicles, and antibody class switching in mouse B cells (59–61). In cGVHD/BO model, the *in vivo* administration of neutralizing anti-IL-17 antibody or small molecule RORγt inhibitors given as a therapeutic markedly alleviated cGVHD (62).

Taken together, our results suggest that targeting the RXR pathway with IRX4204 represents a novel immunomodulatory strategy to prevent or treat cGVHD. IRX4204 is currently in phase I and II clinical trials (NCT0154007 and NCT02991651) to treat cancer. Having shown the beneficial effects of IRX4204 in two distinct cGVHD preclinical models, our studies support consideration for clinical testing of IRX4204 in patients who do not respond to FDA-approved drugs for steroid refractory cGVHD.

## Data Availability Statement

The original contributions presented in the study are included in the article/supplementary material. Further inquiries can be directed to the corresponding authors.

## Ethics Statement

The animal study was reviewed and approved by University of Minnesota.

## Author Contributions

GT designed and performed research, provided, and analyzed the data, and wrote the paper. MZ, FM, RF, JD, SR, MR and EA performed experiments, analyzed the data and edited the manuscript. APM performed histopathological analysis. MS provided IRX4204 and edited the manuscript. BB designed, organized, and supervised research and edited the paper. All authors contributed to the article and approved the submitted version.

## Conflict of Interest

BB receives remuneration as an advisor to Kamon Pharmaceuticals, Five Prime Therapeutics, Regeneron Pharmaceuticals, Magenta Therapeutics, and BlueRock Therapeuetics; research support from Fate Therapeutics, RXi Pharmaceuticals, Alpine Immune Sciences, Abbvie, the Leukemia and Lymphoma Society, the Children’s Cancer Research Fund, and the KidsFirst Fund and is a cofounder of Tmunity. Author MS is shareholder, officer, and director of the company Io Therapeutics, Inc., which is developing IRX4204 for commercialization.The reviewer DW has declared a past co-authorship with one of the authors BB at the time of review.

The remaining authors declare that the research was conducted in the absence of any commercial or financial relationships that could be construed as a potential conflict of interest.

## Publisher’s Note

All claims expressed in this article are solely those of the authors and do not necessarily represent those of their affiliated organizations, or those of the publisher, the editors and the reviewers. Any product that may be evaluated in this article, or claim that may be made by its manufacturer, is not guaranteed or endorsed by the publisher.
